# Retinal degeneration mutation in *Sftpa1*^tm1Kor/J^ and *Sftpd* ^-/-^ targeted mice

**DOI:** 10.1371/journal.pone.0199824

**Published:** 2018-07-03

**Authors:** Faizah Bhatti, Johannes W. Kung, Frederico Vieira

**Affiliations:** 1 Neonatal Perinatal Medicine, Department of Pediatrics, University of Oklahoma Health Sciences Center, Oklahoma City, Oklahoma, United States of America; 2 Department of Ophthalmology and Dean McGee Eye Institute, University of Oklahoma Health Sciences Center, Oklahoma City, Oklahoma, United States of America; 3 Oklahoma Center for Neuroscience, University of Oklahoma Health Sciences Center, Oklahoma City, Oklahoma, United States of America; University of Massachusetts Medical School, UNITED STATES

## Abstract

Surfactant proteins are important collectin immune molecules with a wide distribution throughout the body, including the ocular system. Mice with gene deletions for the surfactant protein genes *Sftpa1* and *Sftpd* were observed to have visual impairment and thinning of the outer nuclear layers of the retina. We hypothesized that gene deletion of *Sftpa1* and *Sftpd* (*Sftpa1*^tm1Kor/J^ and *Sftpd*^-/-^) results in early retinal degeneration in these mice. *Sftpa1*^tm1Kor/J^ and *Sftpd*^-/-^ retinas were evaluated by histopathology and optical coherence tomography (OCT). Retinas from *Sftpa1*^tm1Kor/J^ and *Sftpd* ^-/-^ mice showed early retinal degeneration with loss of the outer nuclear layer. After screening of mice for known retinal degeneration mutations, the mice were found to carry a previously unrecognized Pde6b^rd1^ genotype which resulted from earlier breeding of the strain with Black Swiss mice during their generation. The mutation was outbred and the genotype of *Sftpa1*^tm1Kor/J^ and *Sftpd*^-/-^ was confirmed. Outbreeding of the Pde6b^rd1^ mutation resulted in restoration of normal retinal architecture confirmed by *in vivo* and *in vitro* examination. We can therefore conclude that loss of Sftpa1 and Sftpd do not result in retinal degeneration. We have now generated retinal *Sftpa1* and *Sftpd* targeted mice that exhibit normal retinal histology.

## Introduction

The surfactant proteins A and D (SP-A and SP-D) belong to the super family of collectins or “c-type lectins. Other collectins include mannose binding lectin (Mbl), pulmonary surfactant proteins (SP) SP-A and SP-D, and the conglutinins CL-L1 and CL-P1[1]. The pulmonary surfactant proteins SP-A and SP-D play a key role in pulmonary immunity[2]. They are important in recognizing and mediating neutralization of viruses, bacteria and fungi, clearance of apoptotic and necrotic cells, and resolution of inflammation[3–5]. Recently, these molecules have been found in other organ systems aside from the pulmonary system[6] including vaginal and amniotic fluid, the gastrointestinal tract, renal system and the ocular system[7–15].

Surfactant protein targeted mice, *Sftpa1*^tm1Kor/J^ [4] and *Sftpd* targeted mice (*Sftpd*^*-/-*^*)* [5] were developed by the Whitsett /Korfhagen group at the University of Cincinnati Medical Center in order to study the effect of these proteins in the pulmonary system. Both have been extensively characterized regarding pulmonary phenotype and experiments using these mouse strains have been widely published and cited in the literature. Since their development in the early 1980’s, pulmonary research groups had observed that these mice appeared to have visual disturbances and had blindness (unpublished data), which was attributed to deletion of the surfactant genes. This has never been comprehensively evaluated in the literature. These proteins are very closely associated with the fatty acid pool of surfactant in the pulmonary system. SP-A and D have both been shown to be important in the metabolism of surfactant in that they inhibit of secretion of surfactant from alveolar type II cells, enhance the uptake of diphosphatidylcholine (DPPC) by alveolar cells, they increase the adsorption of phospholipids and help to decrease surface tension in surfactant mixtures[2]. As the rods and cones in the retina have an abundance of fatty acids involved in the signal transduction process, SP-A and or D may impact fatty acids and/or lipase activity in rods and cones of the ONL.

We hypothesized that deletion of *Sftpa1* and *Sftpd* results in early retinal degeneration. Both mouse strains were found to be express the Pde6b retinal degeneration mutation, Pde6b^rd1^ in addition to the targeting of *Sftpa1*^tm1Kor/J^ and *Sftpd*. Our goal is to report the previously unrecognized Pde6b^rd1^ genotype and phenotype in *Sftpa1*^tm1Kor/J^ and *Sftpd* ^-/-^ mice. We report how the baseline retinal phenotype was examined, how the Pde6b^rd1^ genotype was identified and the strategy employed to outbreed this mutation from the mice. We then detail the confirmation of a “wild type” normal retinal phenotype by application of the same rigorous tests.

## Methods

Our experimental approach is summarized here with details following. We first examined and compared the morphology of retinas harvested from *Sftpa1*^tm1Kor/J^ and *Sftpd* ^-/-^ mice to those from wild type C57BL/6J mice by H&E stained sections and *in vivo* using optical coherence topography (OCT). PCR genotyping was performed in order to confirm *Sftpa1* and *Sftpd* knock-down. Genotyping also identified the Pde6b^rd1^ mutation in both strains, *Sftpa1*^tm1Kor/J^ and *Sftpd* ^-/-^. The Pde6b^rd1^ mutation was outbred and retinal morphology was again examined and compared by OCT evaluation as well as by histological examination of retinal cross sections.

### Animals

C57BL/6J wild type mice (WT), and SP-A targeted *Sftpa1*^tm1Kor/J^ mice were purchased from Jackson Laboratories (Bar Harbor ME). *Sftpd* ^-/-^ mice were a kind gift from the Whitsett /Korfhagen lab in Cincinnati, OH. All mice were maintained on a 12-hour light/dark cycle and fed standard mouse chow. The University of Oklahoma Health Sciences Center Institutional Animal Care and Use Committee specifically approved the research protocols associated with this study (IACUC #101341-16-033 and 101005-14-081).

### Examination of microscopic morphology of the retina

Mice (N = 6) were euthanized by CO_2_ asphyxiation. The eyecup was enucleated whole and fixed in PreFer fixative (Anatech Ltd, Battlecreek, MI) for 15 min at room temperature. It was then embedded in paraffin and sectioned at 5 μm onto glass slides. Retinal tissue sections on glass slides were deparaffinized and then incubated with hematoxylin, blue buffer and then incubated with eosin. Microscopic examination was performed using the Olympus IX71microscope (Melville, NY).

### Examination of gross morphology of the retina *in vivo* using OCT

Mice from each strain (N = 6 per strain) were anesthetized with an intraperitoneal injection of a ketamine/xylazine (50–75/1–2 mg/kg) cocktail. Eyes were dilated with tropicamide 1% and phenylephrine 2.5% drops and lubricated with methylcellulose. Spectral-domain optical coherence tomography images were taken with the Envisu R2000 (Bioptigen, Morrisville, NC, USA) at 6 weeks’ postnatal age. Total retinal thickness was measured and compared for all strains by Students *t* test with a significant p value < 0.05.

### Identification of genetic mutation

Mouse genotyping was performed in order to confirm *Sftpa1* and *Sftpd* deletion. Next, a literature review was performed to identify retina mutations known to cause early degeneration in mice. The mutations, retina degeneration 1 (Pde6b^rd1^) and retina degeneration 8 (rd8^-/-^) and Rpe65 gene (Rpe65(450Leu) or Rpe65(450Met))[16] were identified.

### PCR genotyping

Genotyping was performed for the mutations of interest using OneTaq Quick-Load 2X Master Mix (M0486) from New England Biolabs (Ipswich, MA) and oligonucleotides ordered from Integrated DNA Technologies (Coralville, IA). DNA was extracted from ear tissue by boiling it with 50 μL 50 mM NaOH for 60 min at 95 °C, followed by neutralization at room temperature with 25 μL of 100 mM Tris/HCl buffer pH 8.0 containing 10 mM EDTA. 0.5 μL of the supernatant after centrifugation served as DNA template for a 12.5 μL PCR mix. A typical PCR protocol involved an initial denaturing step at 94°C for 30 seconds followed by 30 cycles of 30 seconds at 94°C, 30 seconds at 64°C and 60 seconds at 68°C. The protocol concluded with a final extension at 68°C for 5 minutes. Primer sequences are found in the S1 Table. The primer sequences for *Sftpa1* were taken from the Jackson lab protocols “Sftpa1tm1Kor” (www.jax.org). Integrities of Pde6b^rd1^ and rd8 were verified using the genotyping methods Blazek et al.[17] and of Mattapallil et al.[18]. The RPE65 genotype was confirmed to be solely homozygous Rpe65_450_Met by the method of Grimm et al.[19] using the primers RPE65For/RPE65Rev. The mannose lectin binding (*Mbl*) gene lies in between both the *Sftpa1* and *Sftpd* gene and is also an important collectin protein. In order to confirm that *Sftpa1* and *Sftpd* gene targeting did not disrupt immediately adjacent sequences, the integrity of the *Mbl*1 gene and the 3’-ends of the *Sftpa1* and *Sftpd* genes were further verified by PCR using primers MmMbl1F1/MmMbl1Rev, SPAex6For/SPAex6Rev and SPDex8For/SPDex8Rev.

### Outbreeding the Pde6b^rd1^ mutation

The *Sftpa1* and *Sftpd* mice were mated with C57BL/6J rd1^+/+^ mice (WT mice) in order to outbreed Pde6b^rd1^ from both strains. The F1 generation was genotyped and rd1^+/+^ genotypes of SP^-/-^ as well as SP^+/-^ were selected and mated again for both mutants. *Sftpa1*^-/-^ rd1^+/+^ was confirmed by F4, *Sftpd*^-/-^ rd1^+/+^ was confirmed by F2. The mice were then bred for further six consecutive generations. The resulting genotype was validated for each generation by PCR as well as by histology with cross sections of retina tissues.

## Results

### Retinal morphology in *Sftpa1*^tm1Kor/J^ and *Sftpd*^*-/-*^ mice

**Fig 1:**
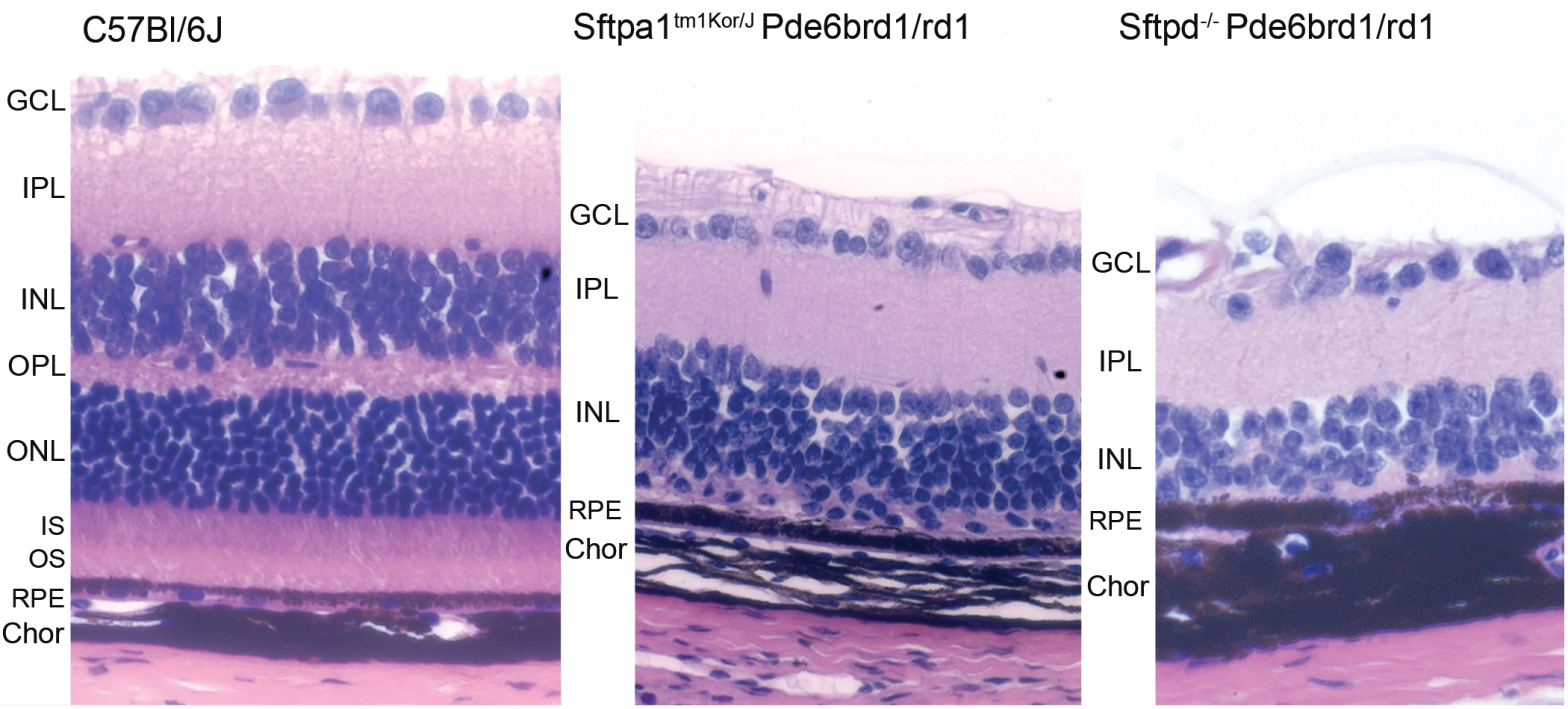
H&E evaluation and comparison of histological morphology of WT C57Bl/6J and *Sftpa1*^tm1Kor/J^ rd1^-/-^ mouse retinas at 3 months of age. The retina from *Sftpa1*^tm1Kor/J^ rd1^-/-^ has attenuation of cellular layers and apparent thickening of the RPE. There also appears to be thickening of the optic nerve layer overlying the ganglion cell bodies. (GCL ganglion cell layer, IPL inner plexiform layer, INL inner nuclear layer, OPL outer plexiform layer, ONL outer nuclear layer, IS inner segments, OS outer segments, RPE retina pigmented epithelium, Chor choroid). Image captured at 40X.

**Fig 2:**
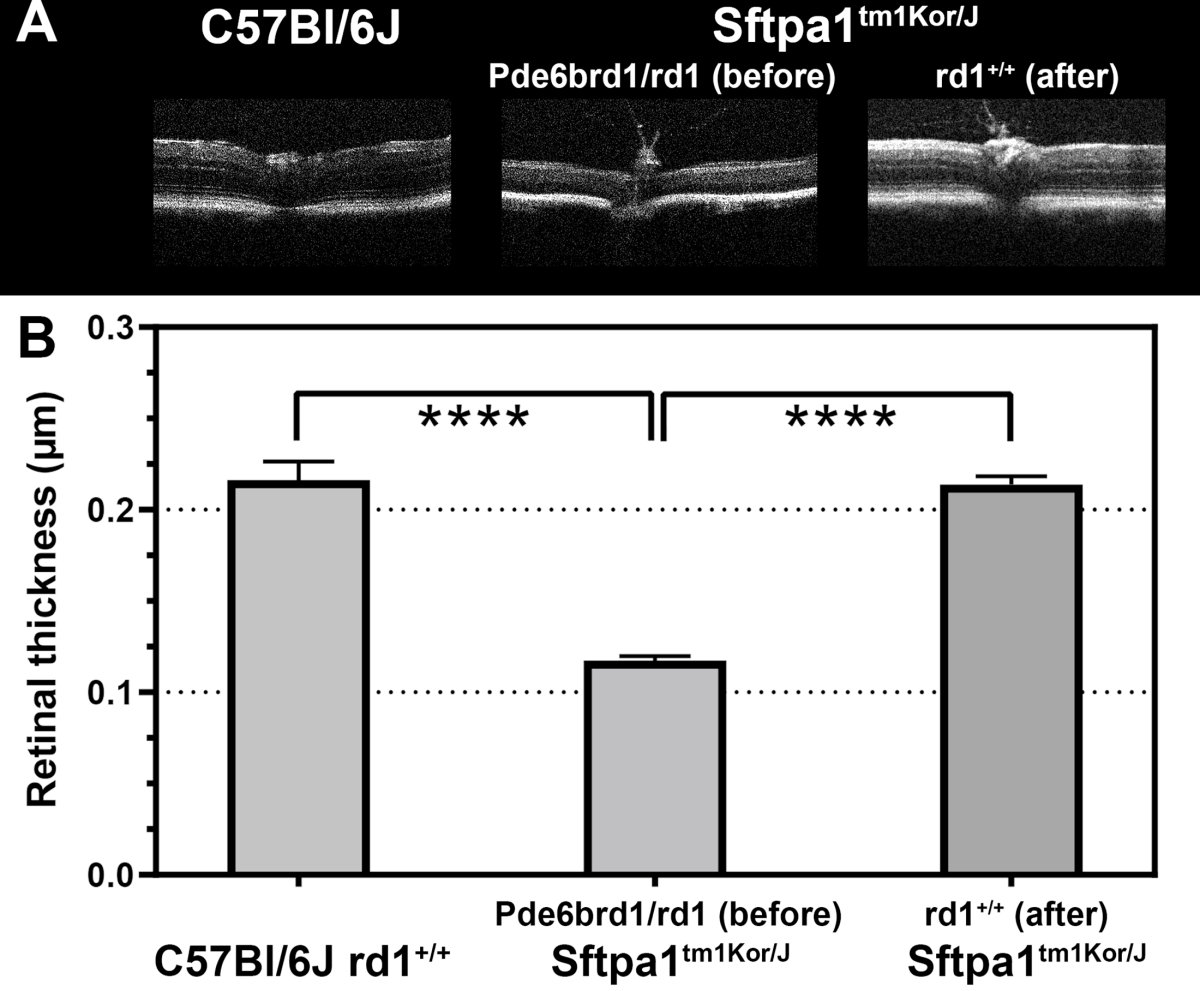
Comparison of retina morphology via SD-OCT (A) and bar graph (B) of 6-week-old WT C57Bl/6J mouse retina and *Sftpa1*^tm1Kor/J^ rd1^-/-^ and the outbreed *Sftpa1*^*tm1Kor/J*^ rd1^*+/+*^. The significant attenuation of retinal thickness as well as obliteration of nuclear layers in the retinas from *Sftpa1*^*tm1Kor/J*^ rd1^*-/-*^ corresponds to a decrease of total retinal thickness by approximately 50%. * *p*-value is 0.0001 (N = 3).

We began our phenotypic assessment by performing microscopic H&E evaluation of retinal cross sections from each group. Retinas from *Sftpa1*^tm1Kor/J^ and *Sftpd*^*-/-*^ at 3 months of age showed diminished retinal thickness as compared to WT C57BL/6J mice. Fig 1 shows the histological morphology as compared by H&E stains. At 3 months of age, loss of the outer nuclear layers (ONL) is observed in both *Sftpa1*^tm1Kor/J^ and *Sftpd*^*-/-*^ strains as compared to WT. We then performed *in vivo* comparison of retinal morphology by OCT, as shown in Fig 2 **panel a)**. OCT evaluation showed a loss of the ONL as well as a significant decrease in total retinal thickness (WT 0.2μm vs *Sftpa1*^-/-^, Pde6b^rd1^ 0.12, p = 0.001) as depicted in the bar graph in Fig 2, **panel b)**.

### Genotyping of mouse strains to confirm mutations

**Fig 3:**
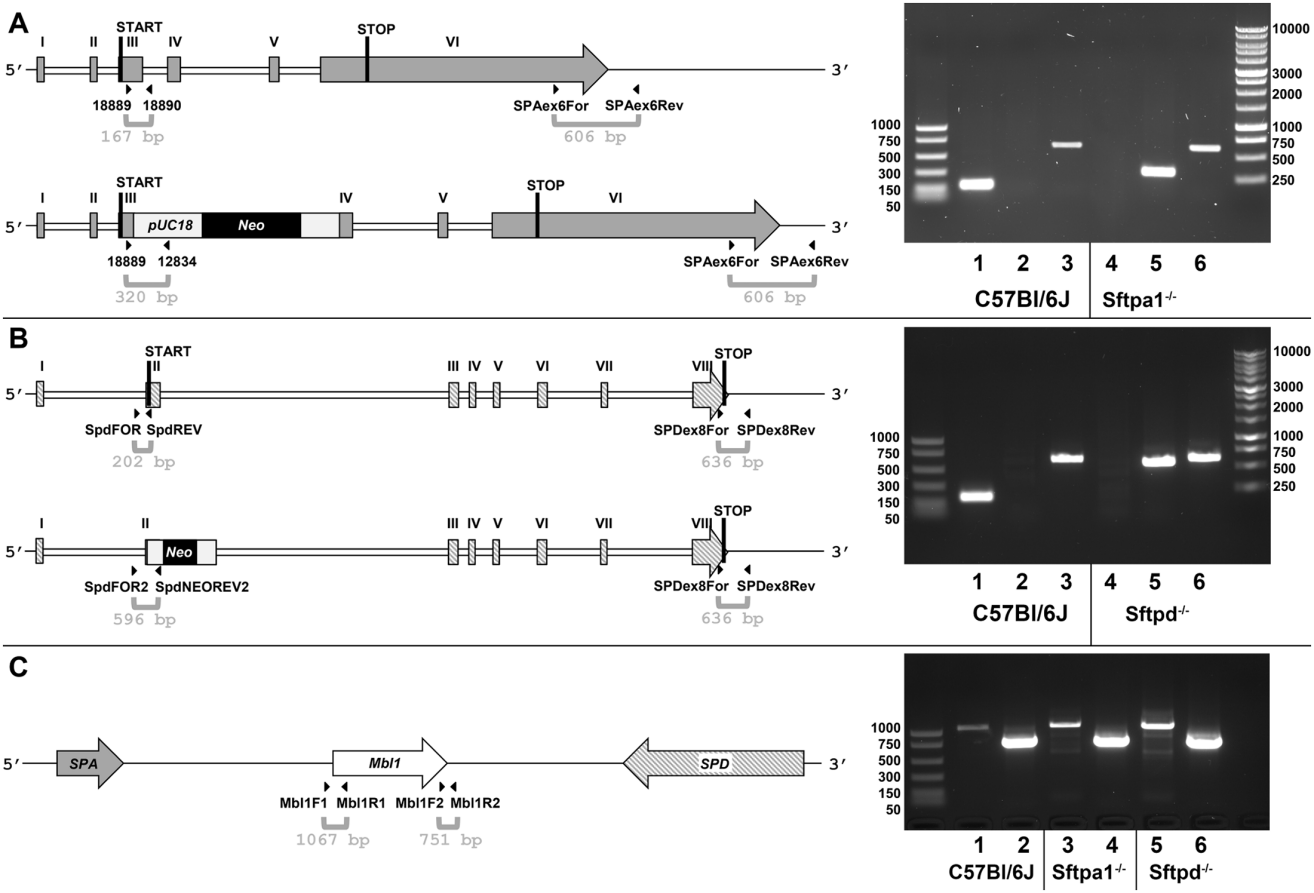
Confirmation of genotype for the *Sftpa1*, *Sftpd* and *Mbl1* gene. (A) Schematic representation of the SP-A encoding gene in wild type (C57BL/6J, upper) and *Sftpa1*^tm1Kor/J^ mice (lower). Exons I-VI with encoded Start and Stop codons of the derived mRNA, primer binding sites and expected fragment sizes for genotyping PCR are shown. Agarose gel with correspondent PCR fragments confirms the two genotypes. DNA from Wild type DNA and *Sftpa1*^tm1Kor/J^ rd1^+/+^ as template were combined with the primer pairs 18889/18890 (lanes 1 and 4), 18889/12834 (lanes 2 and 5) and SPAex6For/SPAex6Rev (lanes 3 and 6), respectively. (B) Wild type gene (upper) and knock out of the *Sftpd* gene (lower). Wild type DNA and *Sftpd*^-/-^ rd1^+/+^ DNA as template were combined with the primer pairs SpdFOR/SpdREV (lanes 1 and 4), SpdFOR2/SpdNEOREV2 (lanes 2 and 5) and SPDex8For/SPDex8Rev (lanes 3 and 6), respectively. (C) Confirmation of the integrity of mannose binding lectin gene in wild type, *Sftpa1*^tm1Kor/J^ and *Sftpd*^*-/-*^ mice. Primer binding sites for *Mbl1* in the gene cluster with the flanking *Sftpa1* and *Sftpd* genes are shown. DNA of wild type, *Sftpa1*^tm1Kor/J^ and *Sftpd*^*-/-*^ were combined with the primer pairs Mbl1F1/Mbl1R1 (lanes 1, 3 and 5) and Mbl1F2/Mbl1R2 (lanes 2, 4 and 6), respectively. Primer sequences are found in the S1 Table.

**Fig 4:**
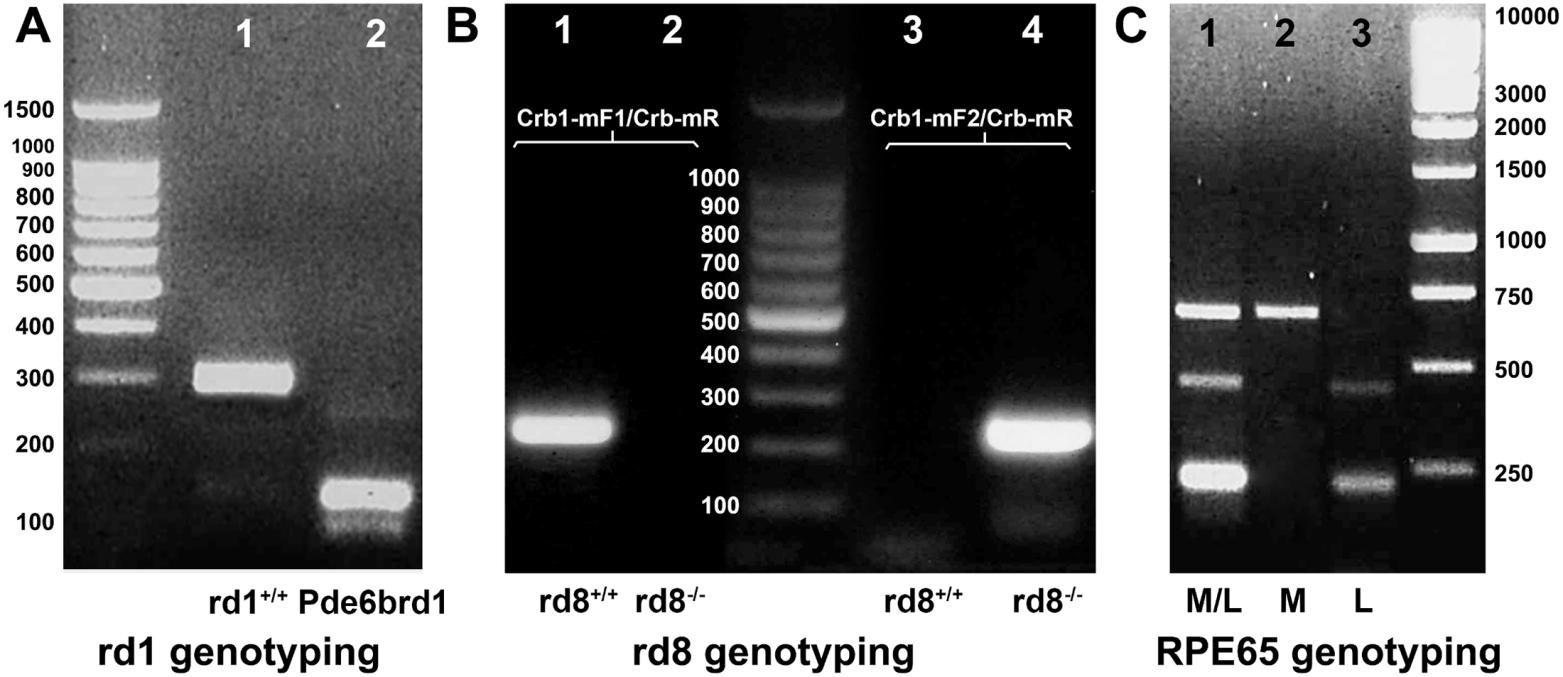
Representative agarose gels with fragments showing genotyping results during outbreeding of the Pde6b^rd1^ mutation in *Sftpa1*^tm1Kor/J^ and *Sftpd* ^-/-^ mice. (A) Retinal degeneration Pde6b^rd1^ with a *DdeI* digest after PCR resulting in specific fragment sizes for the mutated gene. Lane 1: Pde6brd1^+/+^ (298 bp), lane 2: Pde6b^rd1^ (104, and137 bp). (B) Retinal degeneration rd8 genotype: DNA templates with primer pairs Crb1-mF1/Crb-mR for rd8^+/+^ (lanes 1 and 2) and Crb1-mF2/Crb-mR for rd8^-/-^ (lanes 3 and 4). (C) Different RPE65 genotypes: (1) methionine/leucin (2) homozygous methionine (3) homozygous leucine.

The phenotype of the loss of ONL was reminiscent of that seen in mouse strains with early retinal degeneration mutations i.e. those targeting the Pde6b gene. The Pde6b^rd1^ mutation had been reported to occur in C57Bl/6J mice as had the rd8 mutation, therefore, these genetic variants were targeted first. PCR genotyping confirmed that the mouse strains contained the gene manipulation of interest, i.e. *Sftpa1*tm1Kor/J and *Sftpd*^*-/-*^. PCR transcripts targeting the 5’ and 3’-ends of the genes confirmed integrity of the beginning and end of the gene segments as shown in Fig 3a) for *Sftpa1* and Fig 3b) for *Sftpd*. The gene between the two surfactant protein genes encodes the lectin, mannose binding lectin (*Mbl*) known to have similar properties as *Sftpa1* and *Sftpd*, therefore, the 5’- and 3’-ends of the *Mbl* gene were confirmed to be intact as shown Fig 3 **panel c)**. Genotyping for Pde6b^rd1^ is shown in Fig 4. As the rd8 mutation (retinal degeneration 8) and the variant of Rpe65 are also of concern to scientists studying retinal biology, confirmation of genotype was performed. All used mice were found to have the rd8^+/+^ genotype and carry the Rpe65 protein variant with Rpe65_450_Met (methionine at position 450). Representative PCR transcripts are shown in Fig 4.

On successful outbreeding of the Pde6b^rd1^ mutation, final genotypes were [*Sftpa1*^*-/-*^, rd1^*+/+*^, rd8^*+/+*^, *Mbl1*^*+/+*,^ *Rpe65*_450_Me] and [*Sftpd*^*-/-*^, rd1^*+/+*^, rd8^*+/+*^, *Mbl1*^*+/+*,^ *Rpe65*_450_Me].

### Retinal morphology in mice with rd1^+/+^ genotype

**Fig 5:**
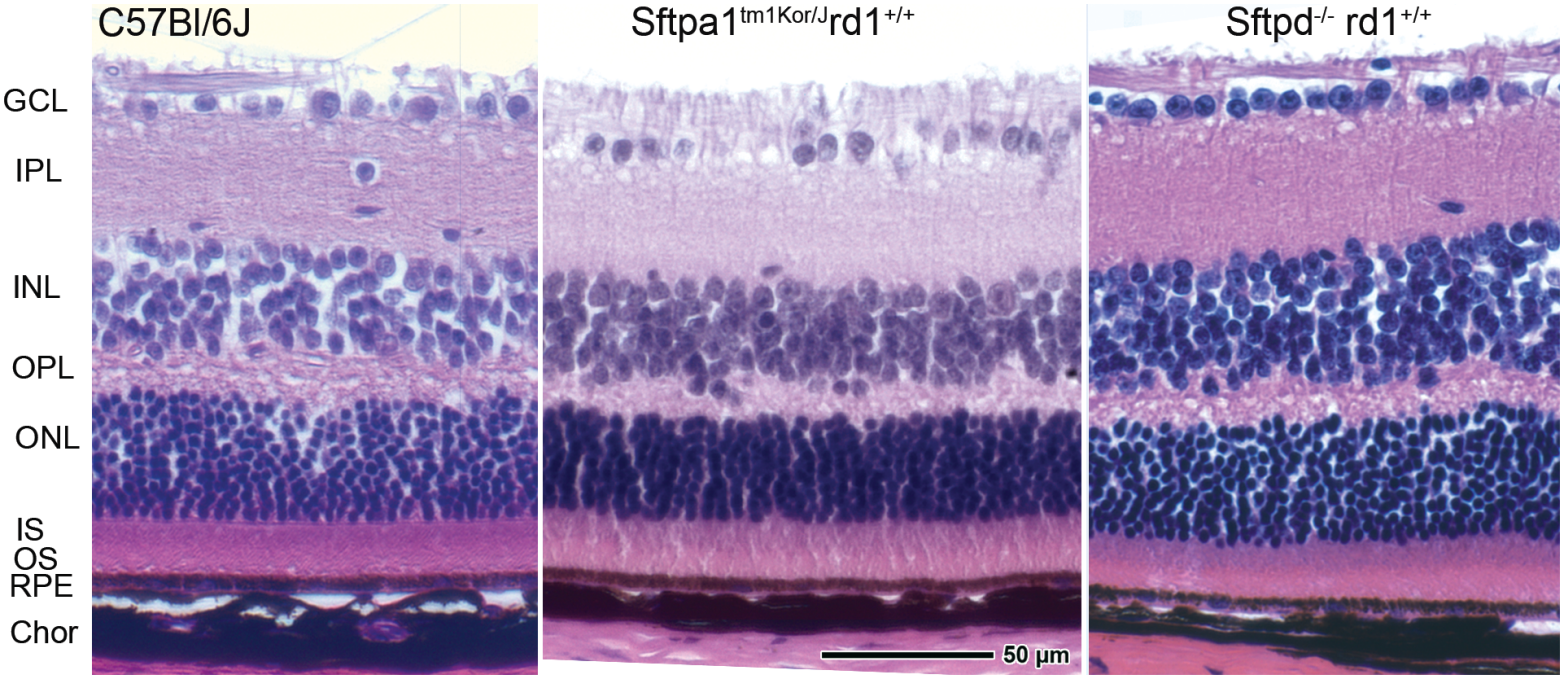
H&E evaluation and comparison of histological morphology of WT C57BL/6J and *Sftpa1*^tm1Kor/J^ rd1^+/+^ mouse retinas at 3 months of age. The outbreeding of Pde6b^rd1^ mutation successfully restores the WT histological phenotype to the mouse retina. (GCL ganglion cell layer, IPL inner plexiform layer, INL inner nuclear layer, OPL outer plexiform layer, ONL outer nuclear layer, IS inner segments, OS outer segments, RPE retina pigmented epithelium, Chor choroid). Image captured at 40X.

Once Pde6b^rd1^ was outbred, we again analyzed the histological archetype of the retinas by H&E stained retinal cross sections in order to confirm if the loss of ONL had been secondary to the Pde6b^rd1^ mutation or if it was secondary to absence of the surfactant proteins. Retinal histological morphology was identical and comparable in [*Sftpa1*^*-/-*^, rd1^*+/+*^, rd8^*+/+*^, *Mbl1*^*+/+*,^ *Rpe65*_450_Me] and [*Sftpd*^*-/-*^, rd1^*+/+*^, rd8^*+/+*^, *Mbl1*^*+/+*,^ *Rpe65*_450_Me] with that of C57BL/6J mice as shown in Fig 5. All strains show normal retinal layer differentiation with a healthy ganglion cell body layer and appropriate nuclear bodies in the inner and outer nuclear layers. The RPE is also of normal appearance. Similarly, OCT also showed restoration of total retinal thickness.

## Discussion

Pulmonary biologists had anecdotally observed that the vision of *Sftpa1*^-/-^ and *Sftpd*^*-/-*^ mice appeared compromised. It was therefore hypothesized that the surfactant gene deletion resulted in retinal abnormalities/degeneration and resultant blindness. We can now confirm that the absence of SP-A and SP-D proteins in ***Sftpa1***^**tm1Kor/J**^ and ***Sftpd*** gene targeted mice, do not lead to early retinal degeneration but is a result of the Pde6b^rd1^ mutation on the Pde6b gene. This retinal degeneration causing mutation was previously unknown to the authors who first described the gene targeted mice, nor to the commercial vendors who now carry these mouse lines. The detection, outbreeding of the mutation, and generation of mice with the said *Sftpa1* and *Sftpd* gene deletion but with intact *Pde6b* gene expression (rd1^+/+^) is reported here. This important finding bears reporting as these mouse strains may be used for study of disease processes unrelated to the pathology for which they were first created.

Our initial hypothesis regarding the potential impact of surfactant proteins A and D on the ONL and rod and cone function was based on the premise that pulmonary SP’s are known to bind to fatty acids including saturated and unsaturated fatty acids of various chain lengths[20]. Furthermore, SP-A has been shown to inhibit phospholipase activity in vitro[21]. Interestingly, in studies employing *Sftpa1*^*-/-*^ mice (*Sftpa1*^-/-^ rd1^+/+^ generated by us), we showed that when retinal architecture is preserved that absence of SP-A protein impacts retinal neovascularization in a mouse model of oxygen-induced retinopathy (OIR)[7]. Thus, it appears that the SP-A protein plays a key role of directing early angiogenic/vascular processes but may not impact the neuronal function of the retina. We were successful in outbreeding the Pde6b^rd1^ mutation, restoring normal retinal architecture that allows us to study the impact of SP-A and SP-D on retinal vascular disease.

Several mouse strains with deletions in pulmonary surfactant proteins have been described in the literature, which are listed in the Knockout Mouse Project Repository (KOMP) and/or in the Jackson Labs Repository. These include the SP-A targeting ***Sftpa1***^**tm1Kor/J**^ mouse, the ***Sftpa1***^**tm2Haw**^ mouse, the ***Sftpa1***^**tm1(KOMP)Vlcg**^ mouse, the conditional cre/lox targeted ***Sftpa1***^**tm1a(KOMP)Wtsi**^. Additionally, a double knockout B6.Cg-*Sftpa1*^tm2Haw^ *Sftpd*^tm2Haw^/J has also been documented. Many studies have been identified in which the ***Sftpa1***^**tm1Kor/J**^ mouse strain was used in organ systems other than the lungs and airways[3, 10, 15, 22]. When generated, these gene-targeted mice are typically characterized regarding the organ system for which they were created. The performance of body wide phenotypic or genotypic analysis is usually not feasible.

In order to determine how the Pde6b^rd1^ mutation may have been introduced in the mouse strains of interest, we reviewed the methods employed by Korfhagen and colleagues in their initial generation of these mice. The SP-A targeted *Sftpa1*^tm1Kor/J^ mouse strain was developed by Korfhagen and colleagues in 1996. The gene targeting strategies of the SP-A^-/-^ and SPD^-/-^ mice have previously been described, in detail[4]. In short, a part of the *Sftpa1* gene was cloned into pUC18 vector and blunt-end ligated with a neo cassette from the vector pGKneoBPA, so that parts of exons 3 and 4 were disrupted. Herpes simplex virus-thymidine kinase (HSV-TK) was cloned into the Puc18 vector downstream of the insert. Embryonic Stem (ES) cells generated from 129-Ola mice were electroporated with the *Sftpa1* targeting construct. Targeted ES cells were selected by culture. Clone 87, a rapidly proliferative, undifferentiated ES cell clone, was microinjected into C57B1/6J host blastocysts. Chimeric males were then bred to NIH Swiss black females. A similar approach was used for generation of *Sftpd*^*-/-*^ mice. The *Sftpd* gene was cloned into the vector with a neo cassette from pGKneoBPA, so that parts of exon 2 and intron 2 were disrupted. These sequences were electroporated onto ES cells and microinjected into C57/B16 host blastocysts. Chimeric males were then bred to NIH Swiss black females. Subsequent breeding was all performed on a C57Bl/6J background, which have also been used as control animals for experiments. Of note, the commercial website (Jax.org) on which these mice are now carried, notes the mouse strain of origin as 129/OlaHsd. Black Swiss mice are not mentioned in the description of the genetics of the mouse. In 2005, it was reported that the Black Swiss and NIH Swiss mouse both had visual and cognitive disturbances which was shown to be secondary to homozygous Pde6b^rd1^ mutation[23]. Several other commonly used mouse strains have subsequently been found to also carry the homozygous rd1 mutation[24, 25].

Mice, homozygous for Pde6b^rd1^ are characterized by early onset, severe retinal degeneration[26, 27]. The *Pde6* gene is important for amplification of visual signals via normal function of the photoreceptors in the retina. The beta subunit (Pde6b) is crucial for proper signaling of the gene[28]. The Pde6b^rd1^ mutation occurs as a result of a murine viral insert[28] and a second nonsense mutation in exon 7 of Pde6b[25]. Yu et al showed that the nonsense mutation, Y347X, was responsible for the clinical presentation of the disease[29].

The importance of thorough analysis of the background of mouse strains and potential for unwanted/unknown mutations is present and risky, as it may compromise the results of all downstream experiments. Unintended and surprise mutations can often be missed. This can sometimes be phenotypically obvious but may be subtle and only apparent at a biochemical level. Some may only appear during developmental stages of the organism (mouse). While genome wide sequencing prior to the use of mice in experiments would be ideal, this is not practical or possible due to the prohibitive high cost. This leaves researchers with a dilemma as there is a risk that experimental results may be tainted due to a mutation or gene variant that has not yet been detected in the strain or has not yet been found to play a role in their field of interest. Caution should be exercised to refrain from over ascribing a phenotype to a particular allele when that phenotype extends beyond the known function of the gene.

In conclusion, the Pde6b^rd1^ mutation is present in *Sftpa1*^tm1Kor/J^ and *Sftpd*^*-/-*^ mice resulting in early, complete retinal degeneration. We were successful in outbreeding of the mutation and were able to confirm normal phenotype in the retinas. The generation of *Sftpa1* and *Sftpd* targeted mice with normal retinal histology will allow us to better characterize the impact of these surfactant proteins on retinal/visual pathways.

## Supporting information

S1 Table Sequences of used oligonucleotides. All the primers (forward and reverse) used for genotyping are listed here with the descriptions of targeted sequences and details of transcripts.
(DOCX)
