# Supplementary material for: Retinal degeneration mutation in Sftpa1tm1Kor/J and Sftpd -/- targeted mice
Source: PLoS One. 2018 Jul 3;13(7):e0199824. doi: 10.1371/journal.pone.0199824 (PMC6029784; doi:10.1371/journal.pone.0199824)
Supplement: S1 Table — All the primers (forward and reverse) used for genotyping are listed here with the descriptions of targeted sequences and details of transcripts. (DOCX) [file pone.0199824.s001.docx]

**S1 Table. Sequences of used oligonucleotides**

| **Primer name** | **Gene** | **5’-Sequence-3’** | **Size of PCR-product** | **Reference** |
| --- | --- | --- | --- | --- |
| 18889 | *SFTPA* | ACAGAAGTTTGTGCCGGAAG | See at 18890 and 12834 | Jackson lab (www.jax.org)  protocol: Sftpa1tm1Kor |
| 18890 | *SFTPA* | ATGGTCACCCAGAAAACAGG | 167 bp with 18889 (in wt) |  |
| 12834 | *SFTPA* | GCTACTTCCATTTGTCACGTCC | 320 bp with 18889 (in SPA^-/-^) |  |
| SPAex6For | *SFTPA* | CTGTTGAGGGTTTGCTGAGAT | 606 bp | (designed for this study) |
| SPAex6Rev | *SFTPA* | TATGTTGATGTACAGTCCTGCA |  |  |
| SpdFOR | *Sftpd* | GGCCAGACCATGTACCTACC | 202 bp | (designed for this study) |
| SpdREV | *Sftpd* | GAGAGAAAGGGCAGCATGTC |  |  |
| SpdFOR2 | *Sftpd* | TGCAGACTCAGCCTCAAATG | 596 bp | (designed for this study) |
| SpdNEOREV2 | *Sftpd* | GGGGAACTTCCTGACTAGGG |  |  |
| SPDex8For | *Sftpd* | ATTGTTCCCTGATGGCCGAA | 636 bp | (designed for this study) |
| SPDex8Rev | *Sftpd* | TGGTAAAACACGGCTCCTCTT |  |  |
| Mbl1F2 | *Mbl1* | CTCAAGCCGAGCATTACGTG | 751 bp | (designed for this study) |
| Mbl1R2 | *Mbl1* | GAGACGCTGGATAGTGGGGA |  |  |
| Mbl1F1 | *Mbl1* | AGAGAACACAGTGGTAGGAAG | 1067 bp | (designed for this study) |
| Mbl1F2 | *Mbl1* | TCCACTCCTACTTTTCCCATTG |  |  |
| Rd1For | *rd1* | CATCCCACCTGAGCTCACAGAAAG | 298 bp for rd1^+/+^  137 and 104 bp for rd^-/-^ | Blazek et al., Dev Dyn 2010^1^ |
| Rd1Rev | *rd1* | GCCTACAACAGAGGAGTTCTAGC |  |  |
| Crb1-mF1 | *rd8* | GTGAAGAAGACAGCTACAGTTCTGATC | 220 bp with Crb1-mR (in rd8^+/+^) | Chang et al., IOVS, 2013^2^ |
| Crb1-mF2 | *rd8* | GCCCCTGTTTGCATGGAGGAAACTTG  GAAGACAGCTACAGTTCTTCTG | 244 bp with Crb1-mR (in rd8^-/-^) |  |
| Crb1-mR | *rd8* | GCCCCATTTGCACACTGAT | See at Crb1-mF1 and Crb1-mF2 |  |
| RPE65For | *RPE65* | CACTGTGGTCTCTGCTATCTTC | 674 bp (methionine variant)  236 and 437 bp (leucine variant) | Grimm et al., J Neurosci 2004^3^ |
| RPE65Rev | *RPE65* | GGTGCAGTTCCACTTCAGTT |  |  |

^1^ Blazek et al., Dev Dyn. 2010 Jun;239(6):1645-53.

^2^ Chang et al., Invest Ophthalmol Vis Sci. 2013 Jul 24;54(7):4974-81.

^3^ Grimm et al., J Neurosci. 2004 Jun 23;24(25):5651-8.
